# Comparative Analyses of Three *Chlorella* Species in Response to Light and Sugar Reveal Distinctive Lipid Accumulation Patterns in the Microalga *C. sorokiniana*

**DOI:** 10.1371/journal.pone.0092460

**Published:** 2014-04-03

**Authors:** Julian N. Rosenberg, Naoko Kobayashi, Austin Barnes, Eric A. Noel, Michael J. Betenbaugh, George A. Oyler

**Affiliations:** 1 Department of Chemical & Biomolecular Engineering, Johns Hopkins University, Baltimore, Maryland, United States of America; 2 Synaptic Research LLC, Baltimore, Maryland, United States of America; 3 Department of Biochemistry, University of Nebraska–Lincoln, Lincoln, Nebraska, United States of America; Center for Nanosciences and Nanotechnology, Mexico

## Abstract

While photosynthetic microalgae, such as *Chlorella*, serve as feedstocks for nutritional oils and biofuels, heterotrophic cultivation can augment growth rates, support high cell densities, and increase triacylglycerol (TAG) lipid content. However, these species differ significantly in their photoautotrophic and heterotrophic characteristics. In this study, the phylogeny of thirty *Chlorella* strains was determined in order to inform bioprospecting efforts and detailed physiological assessment of three species. The growth kinetics and lipid biochemistry of *C. protothecoides* UTEX 411, *C. vulgaris* UTEX 265, and *C. sorokiniana* UTEX 1230 were quantified during photoautotrophy in Bold's basal medium (BBM) and heterotrophy in BBM supplemented with glucose (10 g L^−1^). Heterotrophic growth rates of UTEX 411, 265, and 1230 were found to be 1.5-, 3.7-, and 5-fold higher than their respective autotrophic rates. With a rapid nine-hour heterotrophic doubling time, *Chlorella sorokiniana* UTEX 1230 maximally accumulated 39% total lipids by dry weight during heterotrophy compared to 18% autotrophically. Furthermore, the discrete fatty acid composition of each strain was examined in order to elucidate lipid accumulation patterns under the two trophic conditions. In both modes of growth, UTEX 411 and 265 produced 18∶1 as the principal fatty acid while UTEX 1230 exhibited a 2.5-fold enrichment in 18∶2 relative to 18∶1. Although the total lipid content was highest in UTEX 411 during heterotrophy, UTEX 1230 demonstrated a two-fold increase in its heterotrophic TAG fraction at a rate of 28.9 mg L^−1^ d^−1^ to reach 22% of the biomass, corresponding to as much as 90% of its total lipids. Interestingly, UTEX 1230 growth was restricted during mixotrophy and its TAG production rate was suppressed to 18.2 mg L^−1^ d^−1^. This constraint on carbon flow raises intriguing questions about the impact of sugar and light on the metabolic regulation of microalgal lipid biosynthesis.

## Introduction

While many benefits of microalgae production are inherent to photosynthetic carbon dioxide assimilation, heterotrophic growth can circumvent certain limitations of photoautotrophic cultivation, such as ineffective light transfer in saturated cultures and low photosynthetic efficiencies [1], [2]. During heterotrophy, the presence of a fixed carbon source (*i.e.*, sugar) can promote rapid growth, support high cell densities, and augment lipid accumulation [3]. As such, this mode of growth has been exploited for the industrial production of polyunsaturated fatty acids and bioactive pigments to serve nutritional markets [4], [5]. In recent years, however, incentives to grow oleaginous microalgae have shifted toward biofuels as an end product [6]–[8]. Triacylglycerol (TAG) is a preferred renewable oil because it possesses a high molar ratio of hydrogen to carbon, can be easily extracted from biomass and directly converted to drop-in biofuels by transesterification, hydrotreating, or pyrolysis [9], [10]. If heterotrophic substrates can be sustainably sourced for relatively low cost (*e.g.*, derived from cellulosic biomass, wastewater, or directly produced by other photoautotrophs) [11], the bioconversion of organic compounds to lipids may be a viable model for algal biofuel production [12], [13].

For the commercial cultivation of microalgae, nitrogen limitation is a well studied environmental stressor known to induce lipid accumulation [14]–[17]. Detailed analyses of heterotrophic algal oil production have also been reported and recently reviewed [18]–[23]. Additionally, mixotrophic cultivation with sugar and light can stimulate high oil yields [24]. While we have previously assessed the biomass and lipid productivities of *Nannochloropsis*, *Dunaliella*, and *Chlorella* species grown on a wide variety of sugars [25], the present study focuses on the *Chlorella* genus of green algae *(Chlorophyceae)*, most commonly known for its nutritional benefits when consumed in whole cell dietary supplements [26]–[28]. Various aspects of plant physiology and metabolism have also been studied in *Chlorella* for nearly a century [29], [30]. In the early 1950s, when open pond cultivation systems first became prevalent, *Chlorella* species were some of the first microalgae to be produced in mass quantities and an investigation of scale-up “from laboratory to pilot plant” was reported by Burlew [31].

Despite the long history of the *Chlorella* genus, currently the only species with a fully sequenced, annotated, and publicly available genome is *Chlorella variabilis* NC64A [32]. This unique strain is both the host to large DNA chloroviruses and can be an endosymbiont of *Paramecium bursaria*, surviving through mixotrophic nutrient exchange [33]–[35]. When NC64A is not harbored by *P. bursaria*, it is susceptible to viral infection and requires nutrient supplementation to grow [36]–[38]. While its genome remains a valuable resource for understanding the balance of carbon metabolism, symbiosis, and pathogen-host interactions, NC64A is not a likely candidate for biomass production due to its low yields. Instead, other *Chlorella* species have been cultivated under mixo– and heterotrophic conditions for the production of lutein and astaxanthin (antioxidants) and have served as the basis for mathematical models of sugar-based growth [39]–[44]. Recent metabolic flux analyses and transcriptomic studies performed under different trophic conditions also provide compelling information about shifts in *Chlorella* lipid metabolism [21], [45]–[47].

In order to fulfill an ongoing search for production organisms and model algal systems, the present study assesses the biodiversity of *Chlorella* species based on biofuel production qualities of heterotrophic growth and TAG accumulation when supplemented with glucose at 10 g L^−1^. After phylogenetic sequencing of thirty strains from culture repositories, *C. sorokiniana* UTEX 1230, *C. vulgaris* UTEX 265, and *C. protothecoides* UTEX 411 were selected for comparative analyses based on growth rates, biomass yield, and lipid productivities in photoautotrophic and heterotrophic culture. The influence of heterotrophy and mixotrophy on lipid biochemistry was also investigated through examination of the abundance, composition, and distribution of total oils as membrane-associated lipids, TAG neutral lipids, or accessory lipophilic molecules. Finally, discrete lipid profiles were determined using gas chromatography–mass spectrometry (GC-MS) to evaluate the dynamics of fatty acid chain length and degree of saturation during the course of cultivation. As a result of this comprehensive *Chlorella* species screening, the occurrence of differential lipid compositions led to further consideration of *C. sorokiniana* as a potential platform for bioenergy and biotechnology.

## Results

### Phylogenetic analysis of *Chlorella* species and strain pedigree

**Figure 1:**
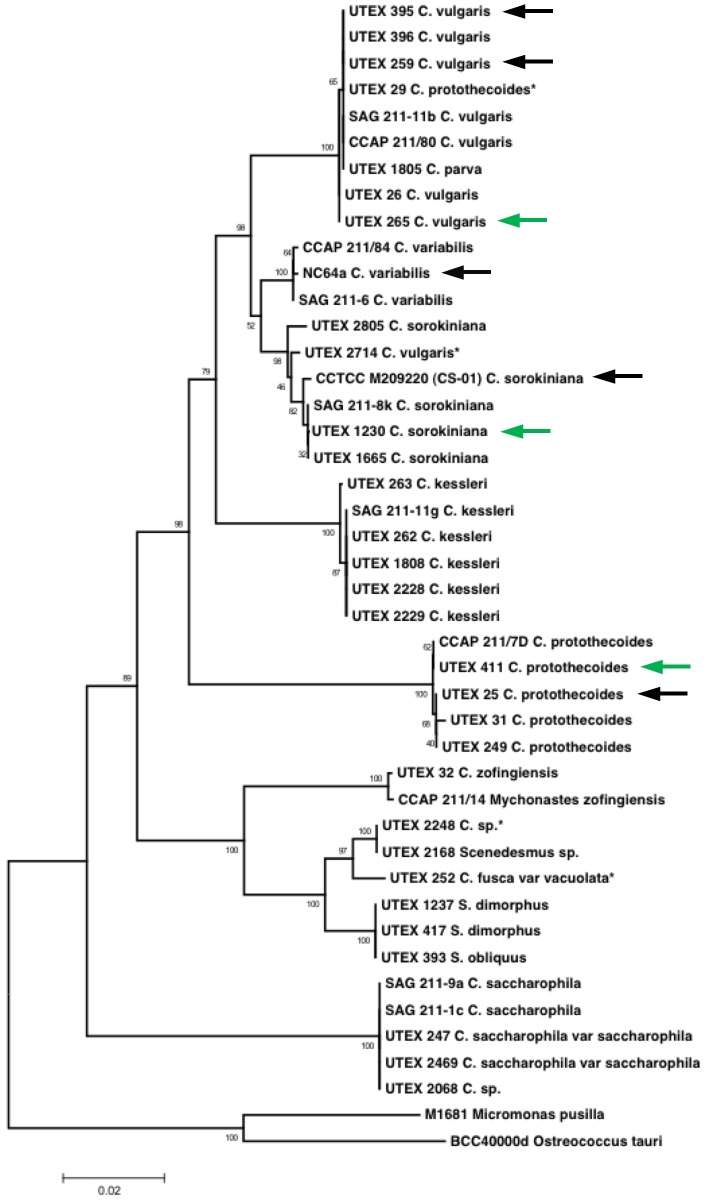
Proposed phylogenetic tree of *Chlorella* species of interest. Green arrows point to branch placement of the three candidate strains examined in this study (*C. vulgaris* UTEX 265, *C. sorokiniana* UTEX 1230, and *C. protothecoides* UTEX 411); black arrows indicate strains with active genome projects. Asterisks denote strains found to align more closely with a species other than their original labeled speciation. The scale bar represents a 2% difference between distinct ITS region sequences.

For our initial species selection, a “genetic fingerprint” based on the 18S ribosomal RNA's internal transcribed spacer (ITS) regions was established for each isolate from a collection of over thirty *Chlorella* strains and used to construct a phylogenetic tree (Figure 1). The annotated 18S ITS sequences for these organisms have been made available online through the GenBank database. During this survey, we encountered some *Chlorella* strains that had been designated as related species (*e.g.*, UTEX 29, 2714) and some strains that group within a separate genus entirely (UTEX 2248, 252). Sequencing of the ITS regions also revealed close phylogenetic relationships between *Chlorella* species in our collection and other strains with active genome projects or available transcriptomes, including NC64A, CS-01, UTEX 395, 259, and 25 [45], [46].

In order to compare autotrophic *Chlorella* species' propensity to grow on glucose and accumulate lipids under uniform heterotrophic conditions, *C. sorokiniana* UTEX 1230, *C. vulgaris* UTEX 265, and *C. protothecoides* UTEX 411 were selected based on vigorous growth attributes during preliminary examination in minimal nutrient Bold's basal medium (BBM) (unpublished data). These three strains were found to be phylogenetically distinguishable within their species groupings and each possesses unique physiological traits. The first in this group, *Chlorella sorokiniana* UTEX 1230, has been studied extensively for its carbohydrate, lipid, protein, and biopolymer content [48]–[50] as well as hydrogen photoproduction [51], glucose transport [52], production of biomolecules by fermentation [53], [54], and bioremediation capabilities [55]–[58]. This organism was isolated by Sorokin and Meyers in the early 1950s [59] and maintained in many culture collections under different strain numbers (originally classified as *C. pyrenoidosa*) [60]–[64].

*Chlorella vulgaris* is a closely related algal species often used as a health supplement [65]. While some annotated genomic sequences have been made available for this species [66], the *C. vulgaris* strain UTEX 265 examined in the present study has not been fully characterized in this manner. Nonetheless, the population dynamics of UTEX 265 have been studied using genetic probes [67] and this particular strain demonstrates heterotrophic growth in medium containing 0.1–0.6% glucose [68]. A related *C. vulgaris* strain (UTEX 395) is the subject of intense transcriptomic and proteomic analyses of lipid biosynthetic pathways [45], [69].

*Chlorella (Auxenochlorella) protothecoides* is a well established heterotrophic alga that can utilize glucose as an organic carbon source [40]. Furthermore, *C. protothecoides* UTEX 411 can grow on sucrose and glycerol [70], [71] and has been exploited for lutein production [40]. Other *C. protothecoides* strains have been cultivated heterotrophically for lipid production— achieving over 55% lipids by dry weight [8] and scale-up to 10,000-L bioreactors [9]. This demonstration has reinforced the potential of heterotrophic *Chlorella* species to generate substantial quantities of lipids for biofuel production.

### Growth and biomass yields of *C. sorokiniana* (UTEX 1230), *C. vulgaris* (UTEX 265), and *C. protothecoides* (UTEX 411)

**Figure 2:**
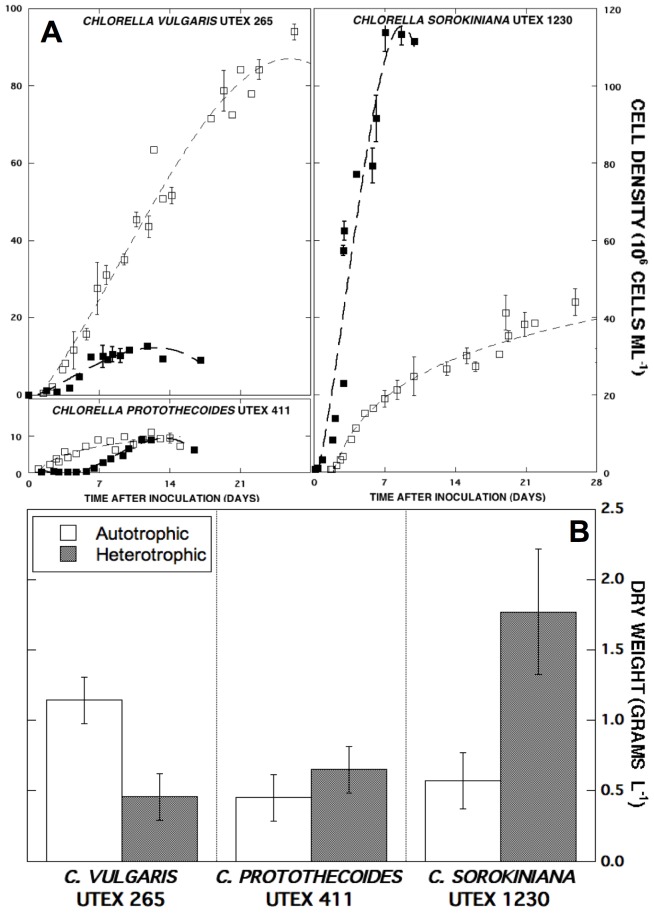
Growth curves and volumetric biomass yields of *C. protothecoides*, *C. vulgaris*, and *C. sorokiniana*. (**A**) Photoautotrophic (□) and heterotrophic (▪) cultures were followed over the course of four weeks or until the population reached stationary phase. Data points are representative of biological replicates and error bars denote standard deviations greater than 1×10^6^ cells ml^−1^. (**B**) *C. protothecoides*, *C. vulgaris*, and *C. sorokiniana* final biomass concentrations in 1.5-L culture during auto– (white) and heterotrophy (gray) are compared. Error bars designate standard deviation from the average of three technical replicates.

The three selected *Chlorella* species were cultivated using previously reported growth methods in separate photoautotrophic and heterotrophic batches for quantitative growth and biomass assessment [72]. The heterotrophic specific growth rates of UTEX 411, 265, and 1230 (0.48, 0.84, and 1.77 d^−1^, respectively) were found to be 1.5-, 3.7-, and 5-fold higher than their autotrophic rates (Table 1). A comparison of auto– and heterotrophic growth curves for *C. sorokiniana*, *C. vulgaris*, and *C. protothecoides* cultures inoculated with 1×10^6^ cells ml^−1^ is shown in Figure 2A. Although heterotrophic growth has been reported for each algal strain [9], [52], [68], [70], [71], *C. sorokiniana* UTEX 1230 demonstrated the most significant heterotrophic advantage in Bold's basal medium with glucose (10 g L^−1^). Within one week, heterotrophic *C. sorokiniana* UTEX 1230 reached the highest cell density of all strains (113×10^6^ cells ml^−1^), while the final heterotrophic cell densities of *C. vulgaris* and *C. protothecoides* cultures were at least 9-fold lower (10−2×10^6^ cells ml^−1^). Under autotrophic conditions, UTEX 265, 1230, and 411 achieved cell densities of roughly 90, 40, and 10 million cells ml^−1^ at their respective stationary phases with comparable photoautotrophic specific growth rates (*μ* = 0.23–0.36 d^−1^).

**Table 1:** Growth characteristics of *C. protothecoides* UTEX 411, *C. vulgaris* UTEX 265, *C. sorokiniana* UTEX 1230.

*Chlorella* Species	*C. protothecoides*	*C. vulgaris*	*C. sorokiniana*
Strain	UTEX 411	UTEX 265	UTEX 1230
Specific Growth Rate, *μ* (d^−1^)	**0.48±0.01**	**0.84±0.09**	**1.77±0.04**
	0.32±0.05	0.23±0.01	0.36±0.05
Doubling Time (hr)	**35±1**	**20±2**	**9±1**
	52±4	72±3	46±3

The major heterotrophic and photoautotrophic growth parameters for each *Chlorella* strain were calculated using data points from the exponential phase of batch culture. Bold values correspond to heterotrophic populations; regular typeface represents autotrophic rates. All kinetic quantities are reported as the average of biological replicates ± standard deviations.

At the termination of each culture, the dry biomass weights of *C. sorokiniana*, *C. vulgaris*, and *C. protothecoides* were compared (Figure 2B). Not surprisingly, heterotrophic *C. sorokiniana* UTEX 1230 amassed the greatest final biomass (1.8 g L^−1^), while the dry weights of *C. vulgaris* and *C. protothecoides* heterotrophic biomass were both close to 0.5 g L^−1^. When grown autotrophically, *Chlorella vulgaris* UTEX 265 reached a final biomass density of 1.1 g L^−1^ (94×10^6^ cells ml^−1^) while *C. sorokiniana* UTEX 1230 reached a maximal cell density of 44×10^6^ cells ml^−1^ at 0.6 g L^−1^ dry weight, similar to the 0.5 g L^−1^ biomass yield of UTEX 411 from only 10×10^6^ cells ml^−1^. Interestingly, *C. protothecoides* UTEX 411 may possess more massive cells based on dry weight although it was unable to reach cell densities above 10 million cells ml^−1^ during either auto– or heterotrophic modes of cultivation in the present study. Both *C. protothecoides* UTEX 411 and *C. vulgaris* UTEX 265 exhibited an extended lag phase during heterotrophy before expansion of the population, suggesting a lengthy acclimation period for growth on simple sugar. Based on its rapid growth and high biomass yield under heterotrophic conditions, *C. sorokiniana* UTEX 1230 was taken into consideration as a lead candidate for an in depth investigation of oil production from heterotrophically-grown cells.

### Lipid content and composition of *Chlorella* during auto– and heterotrophic cultivation

**Figure 3:**
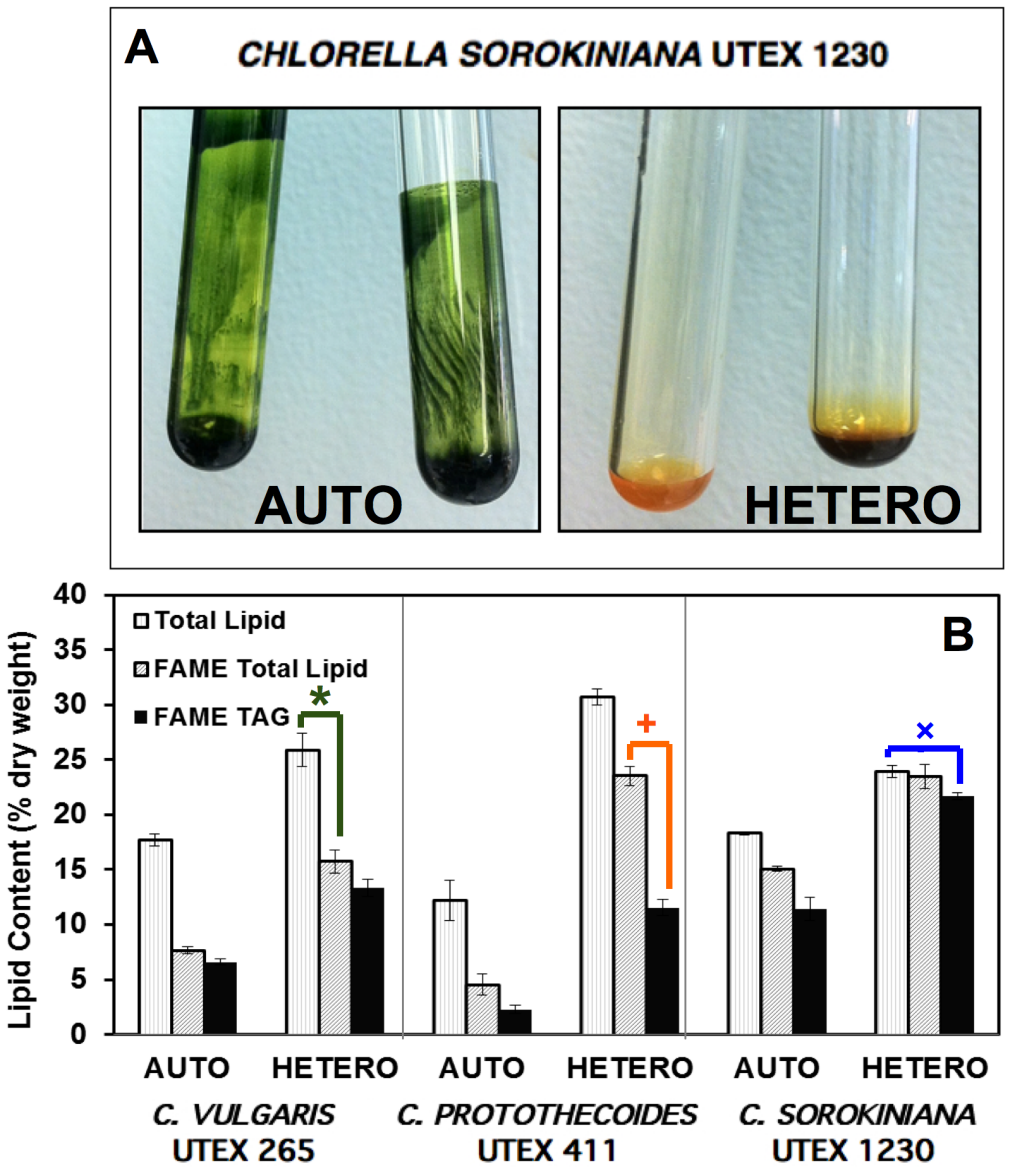
Distribution of total lipid extracts as fatty acids and TAG in three *Chlorella* strains. (**A**) *C. sorokiniana* UTEX 1230 exhibits more prominent chlorosis during heterotrophy than UTEX 265 and 411, which is readily apparent in the pigmentation of total lipid extracts (in color online). (**B**) Total lipids (white) in *C. protothecoides* UTEX 411, *C. vulgaris* UTEX 265, and *C. sorokiniana* UTEX 1230 are classified as FAME (gray) and TAG storage lipids (black). By comparing these values, the relative amounts of supposed accessory metabolites [*] and membrane lipids [+] emerge, while UTEX 1230 appears to be devoid of accessory pigmentation with minimal membrane lipids [×]. Error bars represent one standard deviation from the average of three technical replicates.

In order to evaluate oil production during auto– and heterotrophy, total lipids were extracted from the three *Chlorella* strains followed by transesterification to form fatty acid methyl esters (FAME) for GC-MS analysis. While autotrophic UTEX 1230 lipid extracts remained deeply green due to chlorophyll, heterotrophic lipid extracts exhibited a yellow hue (Figure 3A), indicative of inhibited chlorophyll synthesis. The distribution of each strains' lipid extract is categorized in Figure 3B according to its major components: total lipids measured gravimetrically; total fatty acids measured as FAME; and TAG neutral lipids. In this figure, the inferred difference between FAME (gray bars) and TAG lipids (black bars) in each case is assumed to approximate the amount of membrane lipids [+]. In a similar manner, comparing FAME lipids quantified by GC-MS to total lipid extracts (white bars) leads to the recognition of an unaccounted fraction [*], which represents other lipophilic biomolecules that can be extracted by the organic solvent system, but not measured by GC-MS analysis of the FAME component. This residual fraction is likely to be constituted by chlorophylls, lipid-soluble metabolites, and carotenoids such as lutein. Since many of these accessory “lipids” can be molecules associated with photosynthesis, carbon fluxes are likely to be directed toward these biomolecules rather than TAG during photoautotrophy. For example, UTEX 265 and 411 maintained roughly 60% of their total autotrophic lipids as accessory pigments and metabolites compared to only 16% in UTEX 1230. Consequently, UTEX 1230 partitioned more of its total autotrophic biomass as TAG (11% of the biomass; 60% of total lipids) compared to the markedly lower autotrophic TAG levels in UTEX 265 and 411. The photoautotrophic biomass of *C. sorokiniana* UTEX 1230 was comprised of 18% total lipids, 15% fatty acids, and 11% TAG by dry weight, while *C. vulgaris* UTEX 265 accumulated equivalent amounts of total lipids (18%), but considerably lower levels of fatty acids (7.6%) and TAG (6.6%). *Chlorella protothecoides* UTEX 411 biomass contained the lowest lipid contents during autotrophy with 12% total lipids, 4.5% fatty acids, and 2.3% TAG.

As evidenced by the early onset of chlorosis in *C. sorokiniana* UTEX 1230, accessory pigments can be dramatically reduced during heterotrophy (Figure 3A). Accordingly, this unique phenomenon is manifested in the similarity between the total lipid content and total fatty acid content in UTEX 1230 with marginal amounts of accessory metabolites and minimal membrane lipids [×] (Figure 3B). Since the absence of chlorophyll observed during heterotrophic cultivation of UTEX 265 and 411 was not as severe as UTEX 1230, there is not nearly as much parity between their total lipids and total FAME contents. During heterotrophy, UTEX 1230 total lipid, fatty acid, and TAG contents increased to 24%, 23%, and 21%, respectively, amassing the highest TAG level among all strains examined, which was also two-fold higher than the TAG content of UTEX 1230 during autotrophy. The total lipid, FAME, and TAG contents of heterotrophic UTEX 265 exhibited proportional increases to 26%, 16%, and 13%, respectively. Compared to the other strains, UTEX 411 accumulated the highest total lipid content of 31% with 24% fatty acids during heterotrophy. However, a smaller portion of these lipids was stored as TAG (12% of the biomass; 40% of total lipids). Despite the respectable oil induction in UTEX 265 and 411 with 1.5–2.5 times higher total lipid contents during heterotrophy relative to autotrophy, these two strains possessed slow growth rates and low heterotrophic biomass yields under the current cultivation conditions. Alternatively, UTEX 1230 demonstrated rapid growth during heterotrophy and accrued an overwhelming majority of its total lipid content as TAG.

The quantitative results of neutral lipid induction gathered from UTEX 1230 lipid extracts during heterotrophy were also confirmed with an *in situ* method using Nile Red (NR) to stain whole cells [73]. As conveyed by fluorescent emission curves (Figure S1), heterotrophic UTEX 1230 exhibited a clear maximum of 5.8×10^4^ fluorescent intensity units (FIU) at its expected emission wavelength of 580 nm compared to 2×10^4^ FIU autotrophically. The signatures of these NR profiles indicate that UTEX 1230 accumulated more hydrophobic biomolecules under heterotrophic conditions compared to autotrophy. In addition to different carbon sources, corresponding pH changes in auto– and heterotrophic media during algal growth can also contribute to lipid accumulation and influence fatty acid speciation [74]. While autotrophic cultures of *C. sorokiniana* UTEX 1230 became alkaline with pH rising from 7.3 to 10, heterotrophic cultures experienced slight acidification from pH 6 to 4.8 (Figure S2).

### Distribution of fatty acid chain length and degree of saturation in *Chlorella* species

**Figure 4:**
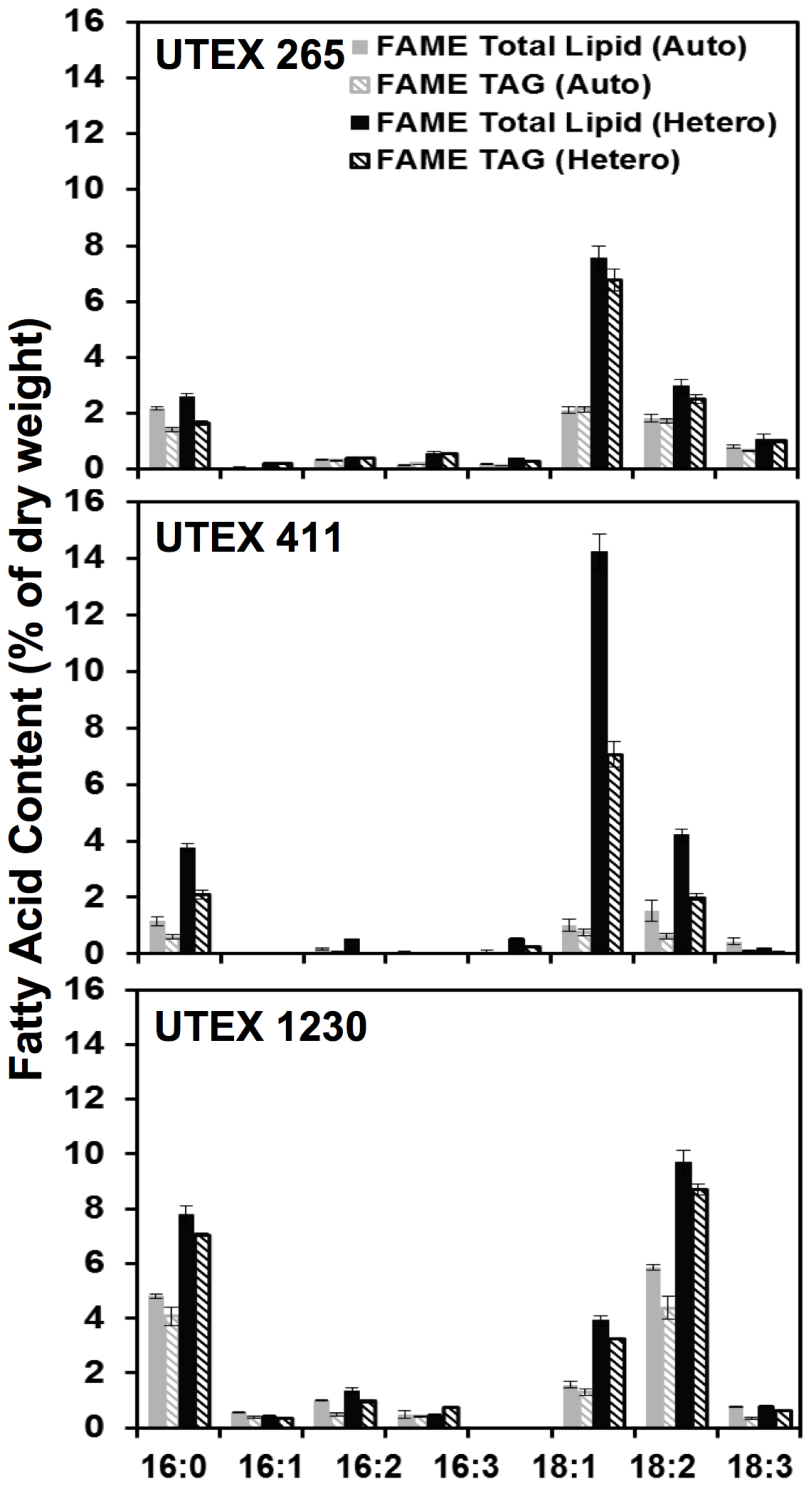
Fatty acid composition of three *Chlorella* strains during auto– and heterotrophy. The distribution of total fatty acids measured as FAME and TAG are plotted as solid and diagonal bars, respectively, for *C. protothecoides* UTEX 411, *C. vulgaris* UTEX 265, and *C. sorokiniana* UTEX 1230. Compared to autotrophy (gray), heterotrophy (black) induces remarkable changes in FAME and TAG lipid profiles. Error bars represent the standard deviation from the average of three biological replicates.

In order to further characterize the lipid metabolic profiles of these three *Chlorella* species, detailed fatty acid compositions were elucidated by GC-MS for auto– and heterotrophy (Figure 4). The typical distribution of sixteen- to eighteen-carbon fatty acids with no more than three degrees of unsaturation (16–18 : ≤3) characteristic of many freshwater green algae was confirmed [74], [75]. While all species exhibited increased total lipid and TAG levels during heterotrophy, Figure 4 highlights the species specificity of fatty acid accumulation with respect to lipid chain length. The discernable differences between the three strains are the ratios of fatty acids accumulated as 16∶0, 18∶1, and 18∶2 as well as the amount of TAG, which is consistent with Figure 3B. During both auto– and heterotrophic conditions, UTEX 1230 showed a relatively even distribution of palmitic (16∶0) and linoleic (18∶2) acids, both between 5–10% by dry weight. In contrast, UTEX 265 and 411 produced oleic acid (18∶1) as the principal fatty acid, reaching heterotrophic maxima of 7.5% and 14%, respectively, with secondary storage lipids of 16∶0 and 18∶2 each at less than 5% of the biomass. Furthermore, UTEX 1230 exhibited greater than 2.5-fold enrichment in 18∶2 relative to 18∶1, which is in direct opposition with the relative levels of oleic and linoleic acids prevalent in the other strains. This polyunsaturation in UTEX 1230 may indicate higher activity of lipid desaturases [76], particularly during heterotrophy [77].

### Time-course comparison of lipid production during hetero– and mixotrophy in UTEX 1230

**Figure 5:**
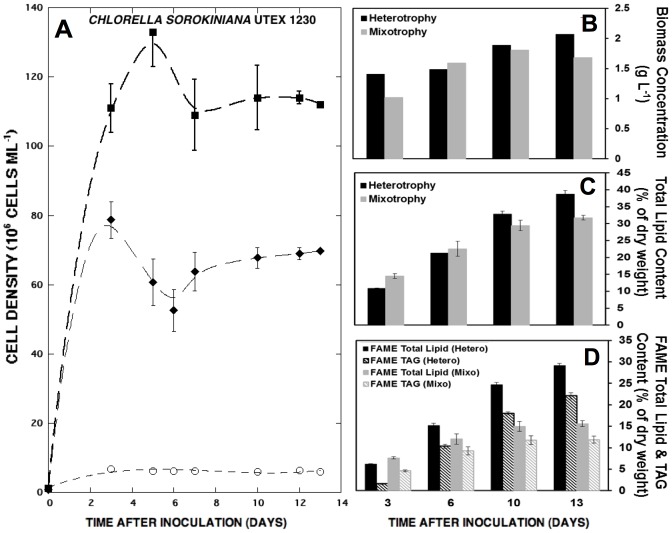
Growth curves, biomass yield, and lipid accumulation of *C. sorokiniana* UTEX 1230 throughout mixo– and heterotrophy in 8-L bioreactors. (**A**) Heterotrophic (▪) and mixotrophic (⧫) cell densities are compared to UTEX 1230 grown in BBM without sugar or light (○), which served as a negative control. (**B**) The corresponding dry biomass, (**C**) total lipid content, and (**D**) total FAME and TAG percentages at days 3, 6, 10, and 13 of mixo– (gray) and heterotrophic (black) cultivation are plotted. FAME and TAG contents for each lipid type are plotted as solid and diagonal bars, respectively. Error bars represent the standard deviation from the average of three technical replicates.

In order to compare the dynamics of lipid production in *C. sorokiniana* UTEX 1230 during hetero– and mixotrophy, cultures were grown in BBM with glucose (10 g L^−1^) either in the dark or together with artificial illumination. Separate 8-L bioreactors were sampled over two weeks for cell density, biomass concentration, and total gravimetric lipid measurements followed by GC-MS analysis. Despite the equivalent amounts of initial glucose, mixotrophic cultures displayed significantly reduced biomass and lipid yields compared to heterotrophic cultures (Figure 5). During the first three to five days of cultivation, both populations expanded at similar specific growth rates; however, clear differences arose later in cultivation (Figure 5A). While heterotrophic growth of UTEX 1230 peaked at 130×10^6^ cells ml^−1^, the mixotrophic culture plateaued at only 70×10^6^ cells ml^−1^, which is more similar to the UTEX 1230 growth pattern during photoautotrophy. The respective hetero– and mixotrophic biomass yields of 2 and 1.7 g L^−1^ were more similar than would be expected from the cell densities (Figure 5B), suggesting a possible photoinhibitory or pH-induced effect on cell cycle progression during mixotrophy, which is also reflected in diminished total lipid content (Figure 5C). Mixotrophic UTEX 1230 biomass contained 31.8±0.7% total lipids after two weeks compared to 38.7±0.9% in the heterotrophic biomass, but experienced a two-fold reduction in TAG accumulation relative to heterotrophy. While mixotrophy engendered a final TAG content of 11.8±0.8% in UTEX 1230, heterotrophy achieved 22.1±0.7% TAG (Figure 5D), corresponding to volumetric TAG productivities of 18.2 and 28.9 mg L^−1^ d^−1^, respectively.

### Progressive induction of polyunsaturated fatty acids in UTEX 1230 during heterotrophy

**Figure 6:**
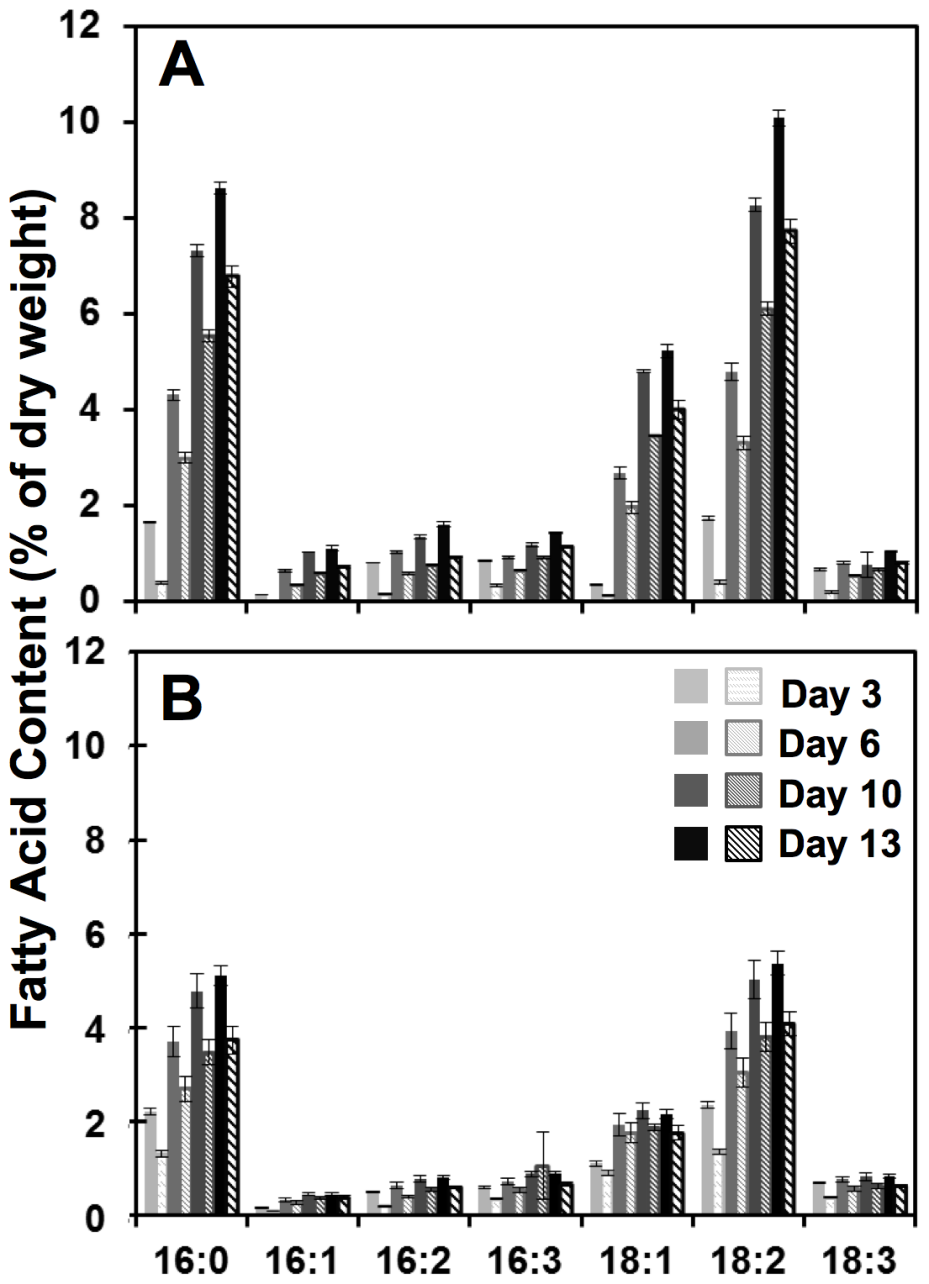
Comparison of total fatty acid and TAG composition of UTEX 1230 during mixo– and heterotrophic cultivation. The discrete lipid profiles of *C. sorokiniana* UTEX 1230 under (**A**) heterotrophic and (**B**) mixotrophic conditions from samples taken on days 3, 6, 10 and 13 of cultivation show fatty acid accumulation rates and patterns. Total FAME and TAG contents for each lipid type are plotted as solid and diagonal bars, respectively. Error bars represent the standard deviation from the average of three biological replicates.

Lipid profiles from this time-course evaluation of UTEX 1230 revealed that the kinetics of fatty acid biosynthesis in heterotrophy are accelerated relative to mixotrophy, especially with regard to the 16∶0–18∶1–18∶2 triad (Figure 6). Time dependent induction of unsaturation led to the biogenesis of 18∶2 (linoleic acid) from precursor 18∶1 (oleic acid) and persisted in late-stage heterotrophic cultures (Figure 6A). In this case, both 18∶1 and 18∶2 showed a steady increase in the first ten days of heterotrophic culture from less than 2% of the biomass (<0.5% TAG) to nearly 5% and 8%, respectively (5–6% TAG). While 18∶1 exhibited an insignificant increase between days ten and thirteen, 18∶2 levels reached 10% of the total dry biomass with 7.7% TAG by the end of heterotrophic culture. Conversely, Figure 6B illustrates that 18∶1 barely exceeded 2% during mixotrophy and 18∶2 reached a peak at approximately 5% dry weight. While total 18∶1 content remained unchanged during the final three days of mixotrophic culture, 18∶1 TAG levels actually dropped while 18∶2 TAG only increased slightly. Interestingly, 16∶0, 18∶1, and 18∶2 TAG fractions had higher initial levels during mixotrophy (closer to 1%), but did not exhibit the high rates of TAG accumulation that occurred during heterotrophy and subsisted at less than 4% of the total biomass by the end of two-week cultivation. Furthermore, the increase in unsaturated 18∶1 and 18∶2 in UTEX 1230 may have been balanced by an enrichment in 16∶0 to decrease membrane fluidity as pH became more acidic during heterotrophy [78].

## Discussion and Conclusions

For the production of microalgal oils, the interdependence of total lipid content, fatty acid composition, and biomass productivity throughout cultivation is critical [79], [80]. Novel approaches to maximize carbon storage as neutral lipids can lead to significant benefits for algae biotechnology, biomanufacturing, and bioenergy [81]. The accumulation of different lipids during heterotrophy and autotrophy can be especially important for the interrogation of lipid biosynthetic pathways in commercially relevant *Chlorella* species. Toward this end, the current study has revealed distinctive auto– and heterotrophic capabilities of three phylogenetically classified *Chlorella* strains with respect to the trade-offs between oil content and growth rate.

### Molecular phylogeny and genomic resources

While microalgae can be generally classified based on cellular features [82], species of the same genus are nearly morphologically identical and can only be definitively differentiated by genotypic analysis [83], [84]. Therefore, the foundation of our species characterization relied on determining the molecular phylogeny of the conserved 18S sequence and species-specific ITS regions for each *Chlorella* strain [85]–[87] employing a rapid colony PCR method [88]. Confirming an organism's true phylogeny is important for understanding what adaptive traits have been collected through natural selection and how they may be useful for biotechnology. In the course of mapping the branches of the phylogenetic tree in this study (Figure 1), the occurrence of mismatched species (*e.g.*, UTEX 29, 252, 2248, 2714) was more prevalent than anticipated. Indeed, another case of species misidentification had previously been identified during genome sequencing of NC64A in tandem with the putative *C. vulgaris* C-169 that is resistant to the *Chlorella* virus [89]. Despite physiological similarities to *C. variabilis*, the C-169 strain was actually determined to be *Coccomyxa subellipsoidea* by comparative genomics [90], [91].

While genetic resources for the *Chlorella* genus are improving with genome sequencing projects underway for many common species [92], the 46-Mb *C. variabilis* NC64A genome is currently the only publicly available *Chlorella* genome [32]. Although peculiar in its growth habits, this organism holds information about the genetic-basis of sugar transport in green algae during symbiosis, which is of particular relevance to the present study [35]. Sequencing of *Chlorella* ITS regions fulfills the need to relate macroscopic growth characteristics of potential production organisms to molecular genetic traits [93]. Thus, the ribosomal ITS-based phylogenetic tree constructed during the present investigation can indicate a strain's proximity to *C. variabilis* NC64A or other organisms with available genetic tools. In addition, this map of phylogeny makes it possible to identify *Chlorella* isolates with suitable biomass and lipid productivities that are both close in genetic relation, yet distinct in physiological characteristics.

### Physiological and biochemical survey of three *Chlorella* species

While the biomass productivity of *Chlorella* cultures has been extensively studied, there remains little consensus between the research community and commercial endeavors regarding preferred *Chlorella* species or growth methodologies to maximize oil yields. Since *C. vulgaris*, *C. protothecoides*, and *C. sorokiniana* are the primary organisms used for process development and strain improvement [94], [95], this work focused on representatives of these species.

In measuring the growth characteristics of *C. sorokiniana* UTEX 1230, *C. vulgaris* UTEX 265 and *C. protothecoides* UTEX 411 (Table 1), UTEX 1230 produced the greatest amount of heterotrophic biomass (Figure 2) in general agreement with previous literature supporting the heterotrophic cultivation of *Chlorella* [96]–[100]. However, *C. protothecoides* UTEX 411 growth did not compare well with the other *Chlorellas* during either hetero– or photoautotrophic modes in our experiments. Compositional differences in media or the effect of pH stress on cell cycle may have accounted for this observed growth restriction, cellular enlargement, and reduced lipid productivity [101]. Compared to BBM used in this study, prior heterotrophic *Chlorella* growth media have sometimes replaced sodium nitrate with glycine and vitamin B_1_ [8], [102], which implicates a potential vitamin requirement or preferred nitrogen source for UTEX 411 [80]. However, we decided to maintain the medium as consistent as possible to limit environmental factors in this comparison. From a technoeconomic perspective, we recognize that the most cost-effective sources and optimal amounts of fixed carbon, nitrogen, and micronutrients may vary for different *Chlorella* species. Furthermore, microalgal strains that can prosper in relatively low nitrogen and carbon concentrations can be desirable for large-scale biomass production by reducing overall nutrient costs and lowering the impact of these resources on bioprocessing costs.

### Lipid allocation between membranes, metabolism, and energy storage in *Chlorella*

One of the main goals of this study was to quantify and compare the differential apportioning of lipid types in the three *Chlorella* strains during auto– and heterotrophy. The divergence in lipid generation between these algal species was clearly evident from the distribution of total lipids, total fatty acids measured as FAME, and TAG neutral lipids. In Figure 3B, heterotrophic *C. vulgaris* UTEX 265 exhibited roughly 10% accessory pigments and lipid-soluble metabolites by dry weight [*], which can serve as high-value nutritional coproducts (*e.g.*, chlorophylls, carotenoids, phenols, tocopherols, sterols). This may indicate that the cellular metabolism of UTEX 265 remains more active than lipid storage during heterotrophy, as observed previously with *C. sorokiniana* strains UTEX 2714 and CS-01 as well as *Chlamydomonas reinhardtii* [56], [103]. In a complementary case, *C. protothecoides* UTEX 411 produced approximately 10% membrane lipids during heterotrophy [+] (*e.g.*, phospho-, galacto-, and sulpholipids). In addition to these detractions from TAG accumulation due to the competing cellular demand for lipid metabolites and membrane structures, both UTEX 265 and 411 exhibited less vigorous heterotrophic growth capacities (Figure 2A). Alternatively, *C. sorokiniana* UTEX 1230 benefited from rapid heterotrophic growth and contained 24% total lipids with close to 90% of these total lipids allocated as TAG [×] (Figure 3B). With energy stores accounting for the majority of UTEX 1230 total lipids, this leaves a limited amount of fatty acids available for membranes during heterotrophy, which may arise from a substantial reduction in the number of chloroplast and thylakoid membranes. Combined with the higher cell densities supported during heterotrophy, UTEX 1230 displayed a 3.8-fold increase in lipids per liter at the end of batch culture relative to autotrophy with an abrupt shift from non-fatty acid lipid production to TAG storage. The increase in pH during autotrophy may be primarily responsible for TAG accumulation due to cell cycle arrest [74]. Alternatively, during heterotrophic cultivation with minor changes in pH, the ultimate lipid yields of UTEX 1230 increased from 0.14 to 0.53 g L^−1^ perhaps due to the up-regulation of stearoyl-ACP desaturase in the presence of glucose, leading to an abundance of 18∶1 precursor fatty acid [77].

Further mixo– and heterotrophic bioreactor optimization at the 8-L scale enabled UTEX 1230 to accumulate 30–40% of its cell mass as lipids, possibly as a result of increased mixing and enhanced gas exchange. Additionally, nutrient utilization trends in previous *Chlorella* studies using BBM demonstrate that nitrate and nitrite levels were reduced to 5–10% of their initial concentrations, indicating that BBM may not be completely deprived of nitrogen at stationary phase [56], [103]. This residual nitrogen may, therefore, sustain the presence of chlorophyll and basal metabolic activity. Furthermore, TAG storage in *Chlorella* can exhibit differential responses to reduced nitrogen levels compared to other algae. While *C. reinhardtii* may require complete nitrogen depletion to induce TAG accumulation [104], *Chlorella* species can store moderate amounts of TAG in nitrogen replete media. This connection between nutrient limitation, cell growth, and lipid biosynthesis offers additional insight into *Chlorella* metabolism.

### Bioenergetics of mixo– and heterotrophic growth and its effect on fatty acid distribution

Considering that the breakdown of sugar by glycolysis may take precedence over carbon dioxide utilization through photosynthesis [72], our study of *C. sorokiniana* UTEX 1230 indeed found no apparent metabolic benefit from light input in addition to glucose. Furthermore, this strain appeared to be potentially maladapted for oil accumulation during mixotrophy. Restricted mixotrophic growth suggests that glucose uptake may be inhibited by light or the energy balance of mixotrophic cells is unfavorably altered (Figure 5A). In extreme cases, pH can also negatively affect sugar uptake via the hexose-proton symporter, although the pH drift encountered in the present study fell within the optimal range for glucose uptake by *Chlorella* (Figure S2) [105]. Interestingly, incongruencies between biomass and cell density in mixo– and heterotrophic comparisons (Figure 5B) also suggest that mixotrophic cells may be larger due to the simultaneous activity of glycolytic lipid accumulation in storage vesicles and photosynthetic carbon fixation, which requires multiple chloroplasts. While these findings contradict a recent report of high mixotrophic productivities from a different *C. sorokiniana* strain, this discrepancy simply highlights the strain-specific metabolic portraits drawn for different *Chlorella* isolates under investigation [106]. Previous studies have indeed found evidence for the inhibition of organic carbon uptake by other *Chlorellas* in the presence of light [107], [108]. In addition, a recent evaluation of *C. sorokiniana* CS-01 (closely related to UTEX 1230) revealed that cytosolic acetyl-coA carboxylase (ACCase) is overexpressed compared to chloroplast ACCase during mixotrophy, suggesting that fatty acid precursors contributing to TAG synthesis are derived from glycolysis of exogenous sugars rather than photosynthetically fixed carbon [72]. This underscores the importance of understanding the bifurcation of carbon utilization during these potentially competing modes of growth.

From the distribution of lipid classes under heterotrophic conditions, it is clear that TAG can constitute a significant portion of lipids in all three of these *Chlorella* strains with 18∶1 and 18∶2 as the primary fatty acids, which are more suitable for biofuel applications than polyunsaturated lipids. From a bioenergetics standpoint, more cellular energy is expended to generate polyunsaturated fatty acids compared to saturated lipids. Since oxidation also has a negative impact on fuel stability, unsaturated lipids must be catalytically hydrogenated prior to fuel blending, which requires additional capital and operating expenses. While the absence of long-chain polyunsaturated fatty acids is not uncommon in *Chlorella* species [109], all strains in the present study exhibited surprisingly low levels of 18∶3 (linolenic acid). Other studies have demonstrated that UTEX 1230 is capable of 18∶3 biosynthesis under autotrophic conditions with nitrogen deprivation [103], [110] and anaerobic digester effluent [56]. In contrast, UTEX 1230 can accumulate higher levels of 18∶1 when supplemented with 3% carbon dioxide (unpublished data).

As a whole, these results support the *Chlorella* genus as a vital source of commercially relevant production organisms with unique lipid metabolic properties, which can vary significantly depending on the presence or absence of sugars and light. While glucose was used in these controlled growth studies, low-cost organic carbon sources are likely to be required for commodity scale biofuel production [56]–[58], [111]. This study's phylogenetic and physiological characterizations of *C. vulgaris* UTEX 265, *C. protothecoides* UTEX 411, and *C. sorokiniana* UTEX 1230 offer insight into the diverse carbon partitioning strategies even within a single genus. In one exemplary case, *C. sorokiniana* UTEX 1230 may serve as a versatile model organism for photosynthetic carbon utilization and heterotrophic lipid biosynthesis with implications for bioenergy and bioprocessing.

## Materials and Methods

### Microalgal cultivation

Stock samples of microalgal strains were obtained from the Culture Collection of Algae at University of Texas at Austin (http://web.biosci.utexas.edu/utex/) and maintained on sterile agar plates (1.5% w∶v) containing Bold's basal medium (BBM) [112]. Cells were cultivated in 1.5-L glass Fernbach flasks or 8-L glass Bellco bioreactors (New Jersey, USA) at 27°C (±1) using BBM. Each axenic batch culture was inoculated with exponentially growing cells at a density of 1×10^6^ cells ml^−1^, constantly stirred, and bubbled with sterile air and monitored with a calibrated M240 digital pH meter (Corning, Inc., New York, USA). Photoautotrophic cultures were illuminated with cool-white fluorescent light at an intensity of 100 µE m^−2^ s^−1^. Heterotrophic and mixotrophic cultures were supplemented with glucose at a concentration of 10 g L^−1^. While heterotrophic cultures were grown in complete darkness, mixotrophic cultures were grown with a light intensity of 100 µE m^−2^ s^−1^. Cell densities were measured by hemocytometer with an Axiovert 100 inverted light microscope (Carl Zeiss, Göttingen, Germany). Measurements were taken in duplicate and experiments were repeated at least three times. Liquid cultures were harvested using a high-speed centrifuge (Sorvall RC-5B, Delaware, USA) at 6,000× *g* for 20 minutes.

### Species identification by genetic sequencing and phylogeny

Species-specific genetic fingerprints were determined by sequencing the internal transcribed spacer (ITS) regions of ribosomal DNA [85]–[87]. Nucleic acids were extracted from clonal algal populations using a 5% Chelex-100 solution as described previously [88]. After boiling at 100°C for 15 minutes, samples were centrifuged at 16,000× *g* for 2 minutes and DNA recovery was quantified using a NanoDrop 2000 spectrophotometer (Thermo Fisher Scientific, Delaware, USA). The primer pair designed for this study (5′-ACTCCGCCGGCACCTTATGAG-3′; 5′-CCGGTTCGCTCGCCGTTACTA-3′) was used to amplify the ITS regions with the Top Taq Master Mix Kit (Qiagen, California, USA) according to the manufacturer's protocol employing thermocycler conditions with an initial melting at 95°C for 2 minutes, followed by 35 cycles of [94°C for 30 sec, 60°C for 30 sec, 72°C for 2 min] and a final elongation at 72°C for 10 minutes. Amplified fragments were separated by electrophoresis on an acrylamide (1% w∶v) tris-borate-EDTA gel. Molecular weights were determined with a GeneRuler 1 kb Plus DNA Ladder (Fermentas, Delaware, USA) and ultimately purified using the GenCatch™ PCR Extraction Kit (Epoch). The resulting nucleotide sequences of 18S ITS regions (Eurofins MWG Operon, Ebersberg, Germany) can be found as annotated entries in the GenBank database (http://www.ncbi.nlm.nih.gov/genbank/) and were aligned using version five of the Molecular Evolutionary Genetics Analysis (MEGA) software suite employing the MUSCLE alignment feature and neighbor joining method to produce phylogenetic trees [113].

### Measurement of algal biomass dry weight and total lipid content

In order to measure dry biomass yield, algal cultures were harvested as described previously, dried overnight in a vacuum oven at 60°C (Precision Scientific Model 5831, NAPCO, Virginia, USA) and weighed in triplicate. For total lipid extraction, washed cell pellets were freeze-dried with a lyophilizer (Model 25 SRC, Virtis, New York, USA) to preserve the integrity of fatty acids and homogenized with a mortar and pestle in liquid nitrogen. For each sample, 100 mg of homogenized biomass was extracted in 6 ml of 1∶2 (v∶v) chloroform∶methanol containing 0.01% butylhydroxyltoluene and 500 µg of tripentadecanoin (15∶0 TAG, Nu-Check Prep, Minnesota, USA) was added as an internal standard [114]. To the 6 ml total extraction volume, 1 ml of 0.7 mm diameter zirconia beads (BioSpec Products, Oklahoma, USA) was added and vortexed at room temperature for 30 minutes. After centrifugation at 1,500× *g* for 5 minutes, the chloroform phase was collected and the water phase was re-extracted using 5 ml of chloroform; the re-extraction was repeated three times. The pooled chloroform phases were evaporated to dryness under a stream of nitrogen. To confirm the methodological validity of manual oil separation [115], total lipid extractions were performed in parallel using an automated Accelerated Solvent Extraction 150 system, which employs elevated temperature and pressure (120°C, 1,500 psi, 4 reflux cycles) to accomplish rigorous oil separation and is generally recognized as the most effective oil extraction equipment (Dionex, Thermo Fischer, Delaware, USA) [116], [117]. The closely comparable total lipid contents of manual and automated extractions were determined in triplicate by gravimetric methods.

### Fatty acid methyl ester (FAME) analysis

Extracted lipids were transmethylated to FAME as previously described [15] and TAG separation was accomplished by thin layer chromatography. FAME was analyzed using an Agilent 6890 Series Gas Chromatography System with an Agilent 5973 Network Mass Selective Detector (Agilent Technologies, Delaware, USA). Chromatography was carried out using a 200 m×250 µm×0.25 µm Varian GC capillary column (Varian Inc., California, USA) with the inlet held at 270°C while 1 µl of the sample was injected using helium as the carrier gas. The oven temperature was programmed for 130°C (10 min) to 160°C (7 min); 160°C to 190°C (7 min), from 190°C to 220°C (22 min) and from 220°C to 250°C (17 min) at a rate of 10°C min^−1^ for each step. The total analysis time was 75 minutes using 70 eV electron impact ionization and data was evaluated with total ion count.

### Relative lipid content analysis *in situ* by Nile Red fluorescence

Nile Red (AnaSpec, Inc., California, USA) was dissolved in acetone to yield a 250× stock solution following previous protocols [73]. For each *Chlorella* sample, 10 µl of Nile Red stock was mixed with 2.5 ml of algal culture diluted to 4×10^7^ cells ml^−1^ in a glass fluorometer cuvette (Starna Cells, Inc., California, USA). A PTI spectrofluorometer (Photon Technology International, Inc., New Jersey, USA) comprised of the SC-500 Shutter Control, MD-5020 Motor Driver, LPS-220B Lamp Power Supply, and 814 Photomultiplier Detection System was used to excite samples at 486 nm and collect fluorescent emission spectra over a 500–660 nm range using the accompanying PTI FeliX32 software.

## Supporting Information

Figure S1 **Nile Red fluorescent emission curves of** ***C. sorokiniana*** **UTEX 1230.** The shifts in emission peaks from UTEX 1230 cultivated under (**A**) autotrophic and (**B**) heterotrophic conditions implicate more significant lipid accumulation during heterotrophy.
(TIFF)

Figure S2 **Change in media pH during auto– and heterotrophic growth of** ***C. sorokiniana*** **.** The pH of auto– (□) and heterotrophic (▪) UTEX 1230 cultures was monitored during exponential growth and remained stable throughout stationary phase. The resulting pH curves demonstrate the interdependence of cell metabolism and the surrounding environment. The standard deviation for each data point is less than 0.5 pH unit (error bars not visible).
(TIFF)
