# Supplementary figures and images for: Comparative Analyses of Three Chlorella Species in Response to Light and Sugar Reveal Distinctive Lipid Accumulation Patterns in the Microalga C. sorokiniana
Source: PLoS One. 2014 Apr 3;9(4):e92460. doi: 10.1371/journal.pone.0092460 (PMC3974682; doi:10.1371/journal.pone.0092460)

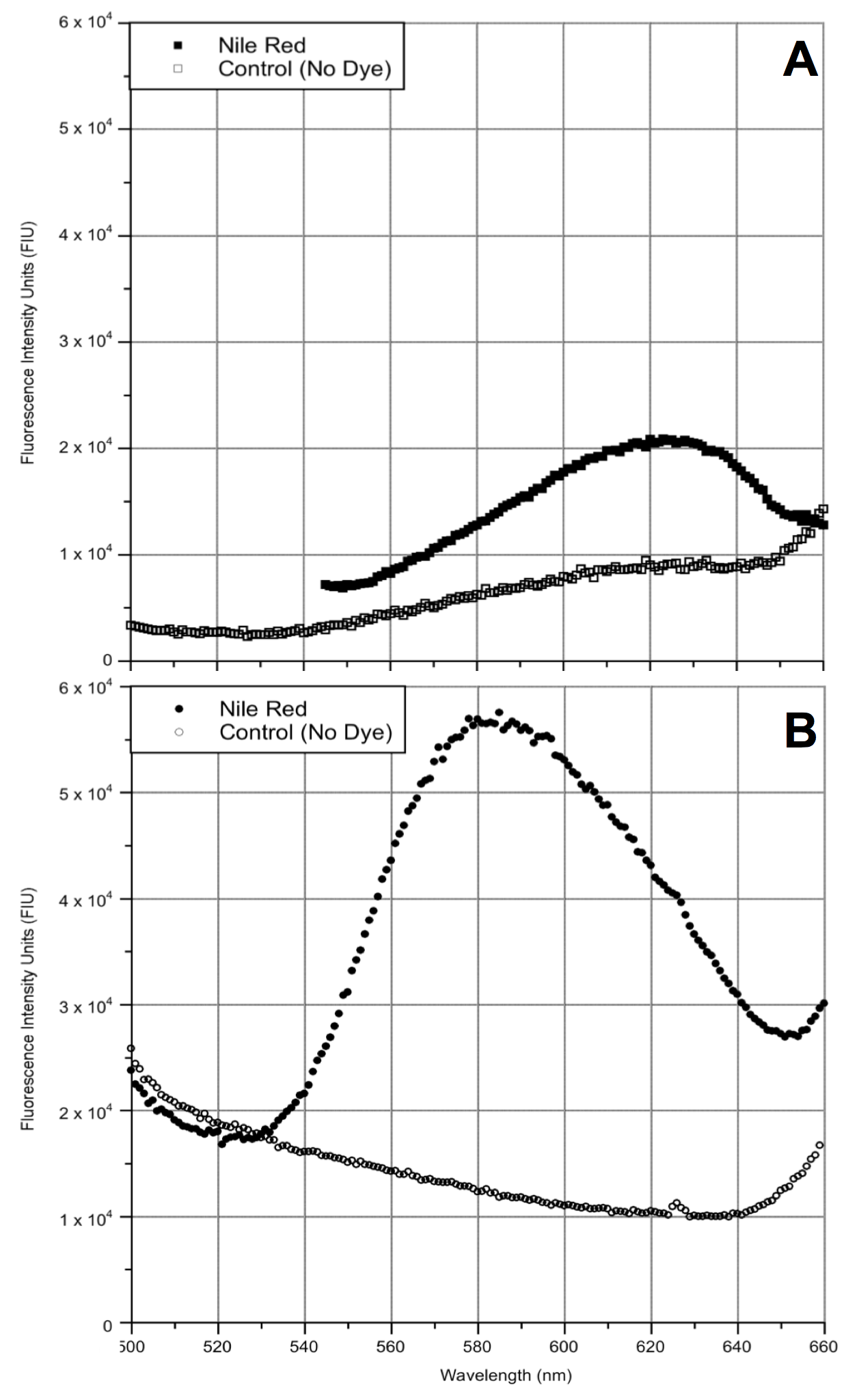

Supplement: Figure S1 — Nile Red fluorescent emission curves of C. sorokiniana UTEX 1230. The shifts in emission peaks from UTEX 1230 cultivated under (A) autotrophic and (B) heterotrophic conditions implicate more significant lipid accumulation during heterotrophy. (TIFF) [file pone.0092460.s001.tiff]

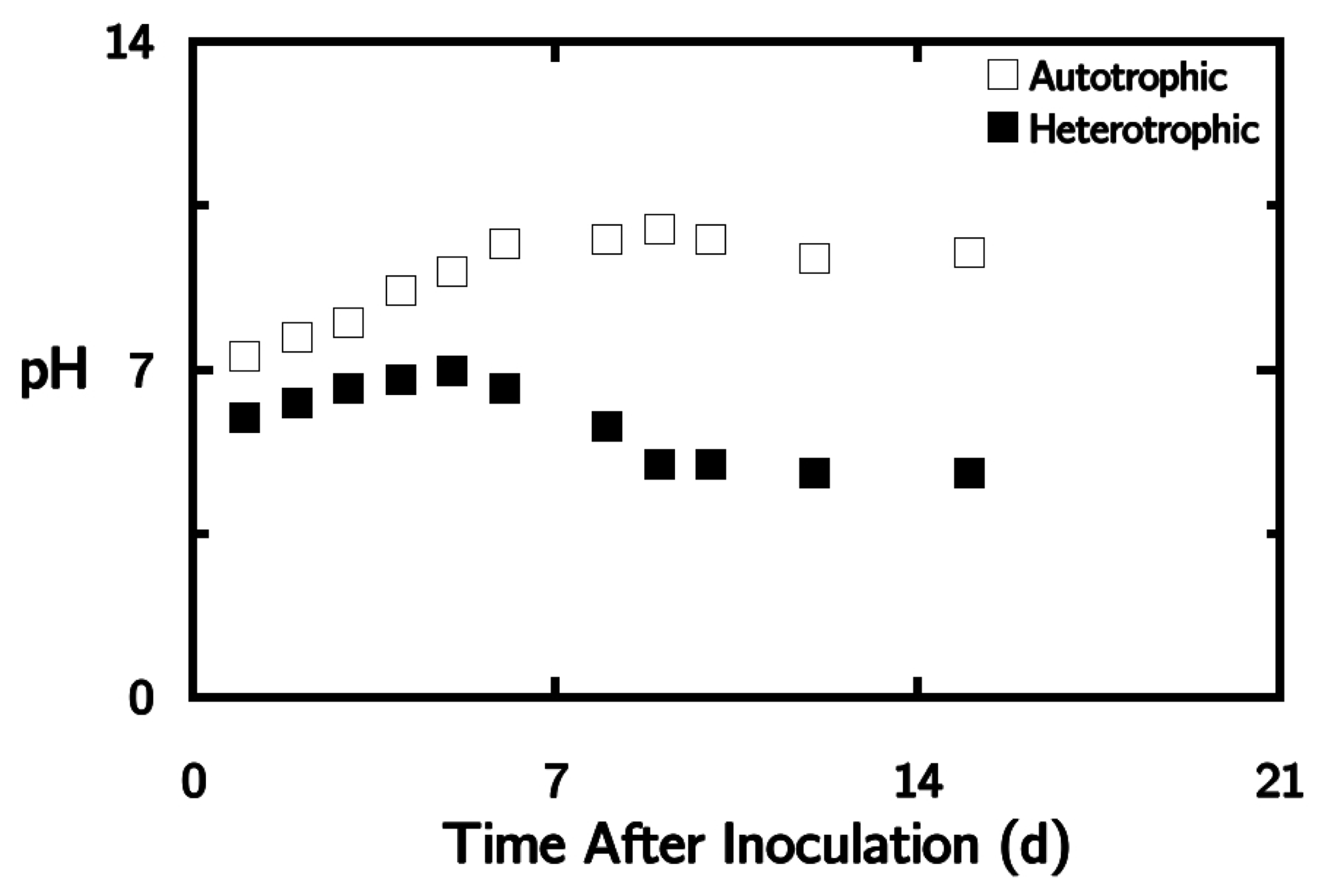

Supplement: Figure S2 — Change in media pH during auto– and heterotrophic growth of C. sorokiniana . The pH of auto– (□) and heterotrophic (▪) UTEX 1230 cultures was monitored during exponential growth and remained stable throughout stationary phase. The resulting pH curves demonstrate the interdependence of cell metabolism and the surrounding environment. The standard deviation for each data point is less than 0.5 pH unit (error bars not visible). (TIFF) [file pone.0092460.s002.tiff]
